# Serosurvey of *Entamoeba Histolytica* Exposure among Tepehuanos Population in Durango, Mexico

**Published:** 2015-06

**Authors:** Cosme Alvarado-Esquivel, Jesús Hernández-Tinoco, Luis Francisco Sánchez-Anguiano, Agar Ramos-Nevárez, Sandra Margarita Cerrillo-Soto, Carlos Alberto Guido-Arreola

**Affiliations:** 1 Biomedical Research Laboratory. Faculty of Medicine and Nutrition, Juárez University of Durango State, Durango, Mexico;; 2 Institute for Scientific Research “Dr. Roberto Rivera Damm”, Juárez University of Durango State, Durango, Mexico;; 3 Clínica de Medicina Familiar. Instituto de Seguridad y Servicios Sociales de los Trabajadores del Estado. Predio Canoas S/N. 34079 Durango, Mexico

**Keywords:** Entamoeba histolytica, seroepidemiologic studies, Tepehuanos, risk factors, Mexico

## Abstract

The seroepidemiology of *Entamoeba histolytica* infection in Tepehuanos population in Mexico is largely unknown. This study aimed to study the seroprevalence and correlates of *E. histolytica* antibodies in Tepehuanos in Durango, Mexico. Through a cross-sectional study, we determined the frequency of *E. histolytica* IgG antibodies in 156 Tepehuanos people in Durango, Mexico using an enzyme-linked immunoassay. Furthermore, we studied the association of *E. histolytica* seroprevalence with the socio-demographic, clinical, and behavioral characteristics of the Tepehuanos studied. Forty-four (28.2%) Tepehuanos with mean age of 31.03 ± 16.71 years old had anti- *E. histolytica* IgG antibodies. Multivariate analysis showed that *E. histolytica* exposure was positively associated with laborer occupation (Odds ratio=2.77; 95% CI: 1.15, 6.66; *p*=0.02), and history of lymphadenopathy (Odds ratio=4.97; 95% CI: 1.74, 14.13; *p*=0.002), and negatively associated with soil contact (Odds ratio=0.13; 95% CI: 0.03, 0.53; *p*=0.004). Other behavioral characteristics including drinking untreated water or unpasteurized milk, and consumption of unwashed raw vegetables or fruits were not associated with *E. histolytica* exposure. The seroprevalence of *E. histolytica* infection in Tepehuanos in Durango is higher thanseroprevalences reported in national surveys. The factors associated with *E. histolytica* seropositivity reported in the present study might aid for the planning and implementation of effective measures against *E. histolytica* infection.

## INTRODUCTION

*Entamoeba histolytica* is a protozoan parasite that causes morbidity and mortality in humans all around the world (1-3). Infection with *E. histolytica* predominates in the developing world where represents an important health problem (4, 5). A major transmission route of *E. histolytica* is ingestion of drinking water or food contaminated with human feces (6, 7). In addition, *E. histolytica* has been responsible of water-associated outbreaks of the disease (8). Sexually transmitted infections with *E. histolytica* have also been reported (9, 10). Most infections with *E. histolytica* are asymptomatic, and infected individuals become parasite carriers (2). However, some individuals may develop a severe disease with hemorrhagic colitis and extra-intestinal disease (11). An important number of travelers suffering from prolonged diarrhea were infected with *E. histolytica* (12, 13). Furthermore, *E. histolytica* is responsible for the development of life-threatening abscesses in organs including liver, brain (14, 15) and lungs (16). Diagnosis of *E. histolytica* infection is based on a combination of serological tests with antigen detection by immunoassays or detection of parasite DNA by polymerase chain reaction (11).

Amebiasis, the disease caused by *E. histolytica*, is recognized as a major source of morbidity and mortality in the developing world (5). However, very little is known about the magnitude of infection with *E. histolytica* in Mexico. In a national serosurvey in this country, researchers found a low (4.49%) seroprevalence of *E. histolytica infection* (17). However, in a recent study in rural population in the northern Mexican state of Durango, researchers found a high (41.8%) seroprevalence of *E. histolytica* infection (18). The seroepidemiology of *E. histolytica* infection in different ethnic groups in Mexico is largely unknown. Tepehuanos is an indigenous ethnic group living in remote mountainous regions in northern Mexico. They live in poverty in rural areas with poor housing conditions and sanitation. These factors may favor transmission of waterborne and foodborne infections among this population including *E. histolytica* infection. In addition, Tepehuanos have limited access to health care facilities and laboratory tests for the diagnosis of *E. histolytica* infection. There are currently no statistics about the magnitude of *E. histolytica* infection in Tepehuanos. This study aimed to determine the seroprevalence of *E. histolytica* IgG antibodies in Tepehuanos population in Durango, Mexico. Furthermore, we sought to determine the socio-demographic, clinical, and behavioral characteristics of the Tepehuanos associated with *E. histolytica* seropositivity.

## METHODS

### Selection and description of the participants

We performed a cross-sectional survey using stored serum samples from a previous study on seroepidemiology of *Toxoplasma gondii* infection in Tepehuanos in Durango, Mexico (19). Serum samples were collected between January 2010 and March 2011. Inclusion criteria for enrollment of the study subjects were: 1) People of Tepehuano ethnicity (indigenous people who speak the Tepehuano language, and identify themselves as Tepehuanos), 2) aged 15 years and older, and 3) who voluntarily accepted to participate in the study, regardless of gender, occupation and socioeconomic status. Exclusion criteria for enrollment of Tepehuanos were: 1) individuals with insufficient amount of serum; and 2) individuals with incomplete epidemiological data. In total, of 180 Tepehuano people invited, 156 agreed to participate in the study.

### General socio-demographic, clinical, and behavioral characteristics of Tepehuanos

We used a standardized questionnaire to obtain the socio-demographic, clinical and behavioral characteristics of the Tepehuanos. A face-to-face interview was conducted to obtain the characteristics of the Tepehuanos. Socio-demographic data including age, birthplace, residence area, educational level, socio-economic status, and employment from all participants were obtained. Classification of socio-economic levels in Tepehuanos was based on their own perception of wealth. Clinical data included health status, presence of gastrointestinal complaints, frequent headache (occurring at least 3 days a week), and history of lymphadenopathy or surgery. Clinical data included in the analysis were those reported by Tepehuanos, and no specific tests to confirm diagnoses were performed. Tepehuanos were asked whether they were aware of suffering from any disease. A Tepehuano was considered as ill when he or she self-reported suffering from any disease either with or without current presence of symptoms. Comorbidities referred by Tepehuanos were diverse and included asthma, diabetes, convulsions, flu, tonsillitis, colitis, cholecystitis and others. Behavioral data included consumption of untreated water or unpasteurized milk, consumption of unwashed raw vegetables or fruits, frequency of eating away from home (in restaurants or fast food outlets), raising farm animals (chickens, cows, horses, goats, or sheep), foreign travel, contact with soil (gardening or agriculture in a regular basis), and type of flooring at home.

### Technical information

Serum samples of Tepehuanos were analyzed for anti-* E. histolytica* IgG antibodies by a commercially available enzyme immunoassay “*E. histolytica* IgG (Amebiasis) ELISA” kit (Diagnostic Automation Inc., Calabasas, CA). According to the kit’s insert, this enzyme immunoassay has a sensitivity of 92% and a specificity of 100%. All assays were performed following the instructions of the manufacturer. Positive and negative controls were run in each assay. We are not aware of further epidemiological studies that had used this kit. Low antibody levels were considered when optical densities between 0.3 to 0.9 were obtained. Whereas, high antibody levels were considered when optical densities higher than 0.9 were obtained.

### Statistics

Results were analyzed with the aid of the software Epi Info version 7 and SPSS version 15.0. We used the Pearson’s Chi-square test and the Fisher exact test, if assumptions for Chi-square test were not met, for descriptive analysis for this study. Multivariate analysis was used to determine the association of *E. histolytica* seropositivity with socio-demographic, clinical, and behavioral characteristics of Tepehuanos. As a criterion to include variables in the multivariate analysis only variables with a *p*≤0.15 obtained in the bivariate analysis were considered. We calculated the odds ratios (OR) and 95% confidence intervals (CI) by using logistic regression analysis. A *p*<0.05 was considered as statistically significant.

### Ethical aspects

In the present study, we used only archival serum samples and data from a previous study (17). The ethics committee of the General Hospital of the Secretary of Health in Durango City, Mexico has approved the previous study. The purpose and procedures of the survey were explained to all Tepehuanos, and a written informed consent was obtained from all of them. In addition, the ethics committee of the Instituto de Seguridad y Servicios Sociales de los Trabajadores del Estado in Durango City approved this project.

## RESULTS

Of the 156 Tepehuanos, 88 were female (56.4%) and 68 were male (43.6%). Mean age of participants was 31.03 ± 16.71 years old, ranging between 15 and 89 years. Forty-four (28.2%) Tepehuanos had anti-* E. histolytica* IgG antibodies. Of these 44 Tepehuanos, 30 (68.2%) had low anti-*E. histolytica* antibody levels and 14 (31.8%) had high anti-*E. histolytica* antibody levels. Socio-demographic characteristics of Tepehuanos and their association with *E. histolytica* seropositivity is shown in Table 1. The variables age and occupation were the only socio-demographic characteristics associated with *E. histolytica* exposure by bivariate analysis. Seroprevalence of *E. histolytica* exposure was higher in laborers than in non-laborers. The frequency of *E. histolytica* exposure was similar among individual occupations.

**Table 1:** Socio-demographic characteristics of Tepehuanos and seroprevalence of *E. histolytica* infection

Characteristic	No. of subjects tested^a^	Prevalence of *E. histolytica* infection		*p* value
		No.	%	

Gender				
Male	68	23	33.8	0.17
Female	88	21	23.9	
Age groups (years)				
20 or less	61	8	13.1	0.003
21-30	38	13	34.2	
31-50	31	15	48.4	
>50	25	8	32.0	
Birth place				
Durango State	153	44	28.8	1
Other Mexican State	2	0	0.0	
Residence area				
Urban	13	2	23.1	0.75
Rural	141	41	29.1	
Educational level				
No education	43	12	27.9	0.52
1-6 years	31	11	35.5	
7 or more years	81	20	24.7	
Occupation				
Laborer^b^	68	25	36.8	0.03
Nonlaborer^c^	88	19	21.6	
Socio-economic level				
Low	147	42	28.6	1
Medium	8	2	25.0	

a Subjects with available data;

b Agriculture, employee, construction worker, business, factory worker, other;

c Housewife or student.

Of the clinical characteristics, only the variables ill status and lymphadenopathy had likely association (*p*≤0.15) with *E. histolytica* exposure by bivariate analysis. Clinical characteristics and a selection of behavioral data of Tepehuanos and their relation with *E. histolytica* seropositivity are shown in Table 2. Of the behavioral data, only the variables raising animals, frequency of eating out of home, consumption of unwashed raw fruits, and soil contact had likely associations (*p* values ≤ 0.15) with *E. histolytica* exposure by bivariate analysis. Results of the multivariate analysis are shown in Table 3. Logistic regression analyses showed that *E. histolytica* exposure was positively associated with laborer occupation (OR=2.77; 95% CI: 1.15, 6.66; *p*=0.02), and history of lymphadenopathy (OR=4.97; 95% CI: 1.74, 14.13; *p*=0.002). In addition, *E. histolytica* exposure was negatively associated with soil contact (OR=0.13; 95% CI: 0.03, 0.53; *p*=0.004).

**Table 2:** Bivariate analysis of clinical and behavioral data and infection with *E. histolytica* in Tepehuanos in Durango, Mexico

Characteristic	No. of Subjects tested^a^	Seroprevalence of *E. histolytica* infection		*p* value
		No.	%	

Clinical status				
Healthy	107	25	23.4	0.08
Ill	46	17	37	
Gastrointestinal complaints				
Yes	9	1	11.1	0.44
No	147	43	29.3	
Lymphadenopathy ever				
Yes	26	14	53.8	0.001
No	130	30	23.1	
Headache frequently				
Yes	65	18	27.7	0.9
No	91	26	28.6	
Surgery ever				
Yes	27	7	25.9	0.77
No	129	37	28.7	
Raising animals				
Yes	130	33	25.4	0.08
No	26	11	42.3	
Eating out of home				
Never	33	14	42.4	0.07
From 1 to 10 times a year	99	22	22.2	
More than 10 times a year	22	7	31.8	
Unwashed raw fruits				
Yes	85	28	32.9	0.11
No	70	15	21.4	
Untreated water				
Yes	128	36	28.1	0.81
No	27	7	25.9	
Soil contact				
Yes	142	35	24.6	0.008
No	13	8	61.5	
Floor at home				
Ceramic	21	4	19	0.54
Concrete	57	18	31.6	
Soil	77	21	27.3	

a Participants with available data.

**Table 3:** Multivariate analysis of selected characteristics of Tepehuanos and their association with *E. histolytica* infection

Characteristic	Odds ratio	95% confidence interval	*p* value

Age	1.07	0.57-2.01	0.8
Laborer	2.77	1.15-6.66	0.02
Raising animals	0.42	0.14-1.22	0.11
Eating out of home	0.93	0.47-1.84	0.84
Unwashed raw fruit consumption	1.41	0.58-3.39	0.43
Soil contact	0.13	0.03-0.53	0.004
Lymphadenopathy	4.97	1.74-14.13	0.002
Ill status	1.07	0.39-2.92	0.89

## DISCUSSION

There is a lack of knowledge about the seroepidemiology of *E. histolytica* infection in ethnic groups in Mexico. Tepehuanos is the most numerous indigenous ethnic group in Durango, Mexico. Therefore, in this study we sought to determine the seroprevalence and correlates of *E. histolytica* exposure in Tepehuanos in the northern Mexican state of Durango.

With respect to seroprevalence, we found a 28.2% seroprevalence of *E. histolytica* infection in Tepehuanos. Remarkably, this *E. histolytica* seroprevalence is higher than other reported *E. histolytica* seroprevalences in Mexican populations. For instance, a low (<5%) seroprevalence of *E. histolytica* was found in a survey in populations living in northern Mexican states (20). However, this previous study was performed about 25 years ago. It is possible that seroprevalence had increased lately. However, interpretation of this comparison should be taken with care since different laboratory tests were used among the studies. A homemade ELISA with 95% sensitivity and 90.7% specificity was used in the previous study (20), whereas a commercially available ELISA with 92% sensitivity and 100% specificity was used in the present study. The seroprevalence found in Tepehuanos is also higher that the 5.95% seroprevalence found in a national survey in 1974 by using counter-current immunoelectrophoresis (21). In addition, the seroprevalence found in Tepehuanos is higher than the 8.41% and 4.49% seroprevalences of *E. histolytica* infection reported in 2 more national surveys reported in the year 1994 by using indirect hemagglutination test (22) and in the year 1995 by using a home-made ELISA (17), respectively. Again, laboratory tests used in the national surveys were different from the commercially available test we used, and comparison of the seroprevalences among the studies should be interpreted with care. It is likely that difference in environment may explain the higher seroprevalence of *E. histolytica* infection in Tepehuanos than in other populations studied in Mexico. Tepehuanos live in rural areas and have a number of contributing factors for infectious diseases as waterborne and foodborne infections. Many Tepehuanos live in poverty, have low education, and suffer from bad nutrition. Their communities have poor sanitation, low housing conditions including poor access to potable water, and lack of sewage systems. The seroprevalence found in Tepehuanos is slightly lower than the 41.8% seroprevalence of *E. histolytica* exposure in general population in rural Durango, Mexico (18). Differences in socio-demographic characteristics of the populations among the studies might explain the difference in the seroprevalence. For instance, the mean age in Tepehuanos was 31.03 years whereas the mean age in general population was 42.91 years (18).

Concerning correlates of *E. histolytica* exposure, a number of contributing factors for *E. histolytica* infection were assessed in the present study. Multivariate analysis of socio-demographic, clinical and behavioral characteristics of Tepehuanos potentially associated with infection by bivariate analysis showed that *E. histolytica* exposure was positively associated with laborer occupation and history of lymphadenopathy, and negatively associated with soil contact. Tepehuanos who worked had a higher seroprevalence of *E. histolytica* infection than subjects who did not work. This finding might suggest that infection was more likely acquired out of home. It is possible that poorer hygiene practices and sanitation existed at their work place than at home. Intriguingly, seroprevalence of *E. histolytica* was higher in Tepehuanos with a history of lymphadenopathy than in those without this history. Lymphadenopathy associated with *E. histolytica* infection has been scantily reported. A case of inguinal necrotizing lymphadenitis caused by *E. histolytica* was reported in the USA (23). General lymphadenopathy in a patient with protracted course of amebic liver abscess, amebic colitis, and fever was reported in Taiwan (24). Infection with *E. histolytica* was linked to the Kikuchi-Fujimoto disease (histiocytic necrotizing lymphadenitis) in a patient in Turkey (25). The association of *E. histolytica* seropositivity and lymphadenopathy found in the present study deserves further investigation. On the other hand, the negative association of *E. histolytica* exposure with soil contact suggest that this factor did not play any contributing role for *E. histolytica* infection among Tepehuanos. We are not aware of further reports about the seroprevalence of *E. histolytica* among ethnic groups. Our seroprevalence results can be a baseline for further research of *E. histolytica* exposure in ethnic groups. In a recent study of general population in rural Durango, Mexico, exposure to *E. histolytica* was associated with housing conditions including source of drinking water and poor education of the head of the family (18). However, these factors were not evaluated in the present study and further research to determine the association of *E. histolytica* exposure with housing conditions of Tepehuanos is needed.

With regard to limitations of the study, the present study has some limitations including a small sample size and limited clinical information. The small size of occupation subsets did not allow us to perform a fairly comparison among individual occupations.

## CONCLUSIONS

We conclude that the seroprevalence of *E. histolytica* exposure in Tepehuanos is higher than *E. histolytica* seroprevalences reported in national surveys. The factors associated with *E. histolytica* seropositivity found in the present study could be useful for the planning of optimal preventive measures against *E. histolytica* infection.
